# Feature Explanations in Recurrent Neural Networks for Predicting Risk of Mortality in Intensive Care Patients

**DOI:** 10.3390/jpm11090934

**Published:** 2021-09-19

**Authors:** Thanakron Na Pattalung, Thammasin Ingviya, Sitthichok Chaichulee

**Affiliations:** 1 Department of Biological Sciences and Biological Engineering, Faculty of Medicine, Prince of Songkla University, Songkhla 90110, Thailand; thanakron.n@psu.ac.th; 2 Department of Familty and Preventive Medicine, Faculty of Medicine, Prince of Songkla University, Songkhla 90110, Thailand; thammasin.i@psu.ac.th; 3 Research Center for Applied Medical Data Analytics, Faculty of Medicine, Prince of Songkla University, Songkhla 90110, Thailand

**Keywords:** critical care, early warning scores, explainable artificial intelligence, machine learning, mortality, time-series prediction, recurrent neural networks

## Abstract

Critical care staff are presented with a large amount of data, which made it difficult to systematically evaluate. Early detection of patients whose condition is deteriorating could reduce mortality, improve treatment outcomes, and allow a better use of healthcare resources. In this study, we propose a data-driven framework for predicting the risk of mortality that combines high-accuracy recurrent neural networks with interpretable explanations. Our model processes time-series of vital signs and laboratory observations to predict the probability of a patient’s mortality in the intensive care unit (ICU). We investigated our approach on three public critical care databases: Multiparameter Intelligent Monitoring in Intensive Care III (MIMIC-III), MIMIC-IV, and eICU. Our models achieved an area under the receiver operating characteristic curve (AUC) of 0.87–0.91. Our approach was not only able to provide the predicted mortality risk but also to recognize and explain the historical contributions of the associated factors to the prediction. The explanations provided by our model were consistent with the literature. Patients may benefit from early intervention if their clinical observations in the ICU are continuously monitored in real time.

## 1. Introduction

Critically ill patients are cared for in the intensive care unit (ICU) with advanced diagnostic and therapeutic technologies. ICU staff are presented with large amounts of clinical data, from which it may be difficult to distinguish the most important features. Delayed identification of patients with deteriorating health and delayed medical interventions could lead to increased morbidity and mortality [1]. Early identification of deteriorating patients could both improve patient outcomes and provide better utilization of healthcare resources.

Predicting clinical outcomes of patients is an important but difficult topic in critical care research. Studies have shown that abnormalities in physiological observations typically occur in patients before major clinical events, such as infection, cardiac arrest, and death. Eighty-four percent of patients exhibited signs of physiological abnormalities within eight hours before the onset of physiological derangement events [2]. Although abnormalities in laboratory results were shown to be inconsistent, abnormalities in vital signs were consistently observed. Many early warning scores (EWSs) have been introduced to provide a bedside assessment system for a patient, such as the Acute Physiology and Chronic Health Evaluation (APACHE) [3,4], the Simplified Acute Physiology Score (SAPS) [5,6], the Modified Early Warning Score (MEWS) [7], the National Early Warning Score 2 (NEWS2) [8], and Between the Flags (BtF) criteria [9]. These clinical scoring systems are mostly based on demographic data as well as vital signs and laboratory values. These scores were based on the studies of aggregated data from large cohorts of patients. They relied on a panel of clinical and statistical experts to develop such scoring systems. Each scoring system was developed for a specific purpose and is considered complementary. The APACHE [3,4] and SAPS [5] scores were developed to assess the severity of the illness affecting patient mortality at 24 h after ICU admission. The MEWS [7] and NEWS2 [8] scores were developed to quickly determine a patient’s level of illness at the bedside. Currently, various EWSs are routinely used in hospitals to identify patients who are likely to deteriorate and to initiate pre-planned escalation of care when needed [10].

The availability of clinical data stored in a modern hospital information system (HIS) enables the development of advanced predictive tools that use statistical techniques and machine learning (ML). These tools could leverage the use of data to identify patterns or relationships between clinical data and patient outcomes. Recently, the Massachusetts Institute of Technology (MIT) Laboratory for Computational Physiology published several public critical care databases [11,12,13] to promote the development of machine learning algorithms on large datasets. This has led to the development of various data-driven approaches for EWSs. Previous studies have used descriptive statistics on clinical observations and tree-based algorithms for mortality prediction [14,15,16]. Several studies employed time-series techniques to process real-valued clinical observations for mortality prediction [17,18,19,20,21]. Some studies have developed algorithms that can predict multiple important clinical events [22,23,24]. Most studies demonstrated their algorithms performed better than the traditional scoring systems used in the ICU.

Although ML models have demonstrated their robustness in various domains, their decisions are still not transparent. Many questions have been raised about the trust in the model and the adequacy of its predictive performance. Methods used for model interpretation often focus on explaining the model by examining the contribution of each feature to the output of a model. Recently, Lundberg et al. [25] introduced SHapley Additive exPlanation (SHAP), which uses Shapley values to provide local explanation ability to the model. A Shapley value represents the average contribution of a feature over all possible combinations with other features. SHAP uses these Shapley values to measure the effect of each individual feature on a single prediction. This involves permuting all combinations of the removal of a feature to determine its effect on the prediction. The visualization of contribution scores could be thought of as a tool for clinicians to facilitate their decision-making process [26].

This study aims to apply recurrent neural networks (RNN) with explainability to predict the mortality risk in intensive care patients. The explainability method enables causal explanation behind the deep learning model so that a prediction made by the black box model becomes visible. Our proposed approach processes time-series of vital signs and laboratory observations for predicting a risk of mortality, so the score indicating the severity of illness could be given. We employ SHAP to explain a model’s decision on time-series data such that contributions of each variable for each time window could be determined and clinical variables of high importance could be highlighted. We investigate our approaches on three recent RNN architectures, namely fully-connected RNN (FRNN), long short-term memory (LSTM), and gated recurrent unit (GRU). We evaluate our techniques on three public critical care databases, namely Multiparameter Intelligent Monitoring in Intensive Care III (MIMIC-III) [11], MIMIC-IV [12], and eICU [13] databases.

Our contributions are three-fold. First, we present detailed results of different RNN algorithms. Our results are consistent with the associated clinical events (ICU mortality and surviving to ICU discharge). Second, we calculate feature importance for both variable and time dimensions through SHAP. The important features we identified are consistent with the factors associated with the clinical deterioration and mortality described in the literature. Finally, we employ the same standardized methods and evaluate our approaches on three critical care databases to obtain detailed benchmarking results. We emphasize that our methodology is robust, and our results are similar across different critical care databases.

This paper is organized as follows. Section 2 reviews related literature that serves as a frame of reference for this study. Section 3 describes the critical care databases used in our study, explains the data preparation steps, and details the methodology used to develop and evaluate our prediction algorithms. Section 4 details the comparative results for each critical care database. Section 5 discusses our results, compares them to other related studies, and provides an analysis of feature importance. Section 6 summarizes the main findings of this study.

## 2. Related Work

Patients admitted to the ICU usually suffer from multiple diseases and require close and continuous monitoring with extensive equipment to prevent the possible rapid deterioration of their health. This results in extensive clinical data that require efficient and accurate systems to support data analysis. The use of critical care data to predict future events, i.e., patient mortality, is considered one of the most important topics in critical care research. This section reviews the relevant literature on this topic, focusing on conventional scoring systems and ML approaches for mortality prediction.

### 2.1. Conventional Scoring Systems for Mortality Prediction

Manual surveillance of possible abnormalities in patients may not be effective enough to help patients in a timely manner. Therefore, standards for surveillance and follow-up with the patients have been proposed. Several EWSs were developed as general physiological bedside assessment systems to assist in monitoring patient changes during hospitalization [7]. APACHE scores [3,4] are one of the tools mostly used to predict patient mortality following ICU admission. The scores are based on various clinical variables, including vital signs and laboratory values, obtained during the first 24 h of ICU admission. There are several versions of APACHE scores: APACHE-II [3] (12 variables), APACHE-III [4] (20 variables), and APACHE-IV [27] (27 variables). APACHE-II and APACHE-III were primarily developed to predict mortality in septic patients. APACHE-IV [27] was developed specifically for predicting short-term hospital mortality in general critically ill adults. APACHE-IV reported an AUC of 0.88 but with a larger number of clinical variables compared to the older releases. SAPS scores [5,6] are also scoring systems used for specifying the severity of illnesses in intensive care patients, which, in turn, could be used to estimate mortality similar to APACHE scores.

The conventional point-based scoring systems are based on a certain set of vital signs, laboratory values, and clinical details. Although they have been reported to have weaknesses in discrimination, there is currently no up-to-date scoring system that can be readily and efficiently used for mortality prediction in the clinic. With so much clinical data available in HIS, the need for other techniques that can assist in the early prediction of mortality has increased.

### 2.2. ML Approaches for Mortality Prediction

ML algorithms can be applied to clinical data stored in an HIS to predict important clinical events, disease progression, and treatment outcomes. ML examines the relationships between clinical variables and patient outcomes, and then creates a data-driven approach to predict future outcomes. A number of studies have investigated the prediction of mortality in critical care patients. Most studies used large critical care databases provided by the PhysioNet repository to develop such algorithms.

Several studies employed descriptive statistics to calculate simple summaries (e.g., minimum, mean, maximum) for the clinical measurements made within the time window (e.g., 24/48 h) and classical ML techniques (e.g., logistic regression and decision trees) to develop a model [14,15,16]. Awad et al. [14] used descriptive statistics of the measurements taken during the first 6 h after admission and developed a tree-based method for mortality prediction in ICU patients with an AUC of 0.82. A similar approach was taken by Johnson et al. [15], who calculated the descriptive statistics from the measurements in a 24-h window and developed the model using a tree-based gradient boosting algorithm with an AUC of 0.92. The authors included the components of the Glasgow Coma Scale (GCS), which are the nurse assessments (motor, verbal, and eyes) of impairment of consciousness in response to stimuli, in the model. Recently, El-Rashidy et al. [16] developed an ensemble method based on five tree-based techniques using the data statistics of over 80 of the most important clinical variables calculated based on a 24-h time window. The authors achieved an AUC of 0.93. Most of the models that performed best were based on tree-based classifiers. The more clinical variables included, the better the results.

Currently, many studies explored ML techniques without feature engineering, i.e., with real-valued time-series measurements. Desautels et al. [17] developed an ensemble of tree-based classifiers for mortality prediction with hourly time-series measurements of 14 clinical variables over a 5-h period. The authors achieved an AUC of 0.78 on MIMIC-III. Caicedo-Torres et al. [20] proposed a one-dimensional convolutional neural network (CNN) that takes 48-h time-series measurements of over 22 clinical variables for mortality prediction. Their CNN attained an AUC of 0.87. Ge et al. [18] developed an LSTM model for mortality prediction on their institutional critical care databases. Their model takes hourly measurements over a 48-h period and achieved an AUC of 0.76. Jun et al. [21] proposed a bidirectional GRU model for mortality prediction that takes bi-hourly measurements of 99 clinical variables over a 48-h period. Their model achieved an AUC of 0.87. Due to the high percentage of missing values, it may be difficult for the models to learn the temporal dynamics contained in real-valued time-series measurements.

Recently, the advantages of modeling with time-series measurements were highlighted by Lundberg et al. [25], who proposed SHAP to explain the model by calculating the contribution of each clinical variable at each time step to an expected outcome. The resulting contributions could provide general insights into the precise changes in risks caused by specific patient characteristics. Lauritsen et al. [23] used SHAP to explain the risk of acute critical illness (e.g., sepsis, acute kidney injury, and acute lung injury) by pinpointing important variables and temporal features that are essential to a particular outcome, ensuring clinicians can understand the reasoning underlying the prediction.

## 3. Materials and Methods

This section describes the research methodology used to develop our algorithms. We followed the five-step SEMMA (Sample, Explore, Modify, Model, and Assess) approach [28]. Section 3.1 describes the selection of study cohorts from each critical care database. Section 3.2 examines patient demographics and clinical variables in relation to mortality in critical care patients. Section 3.3 explains the pre-processing of clinical variables and the preparation process for data modeling. Section 3.4 describes the formulation of the study problems and the process by which the models were developed. Section 3.5 concerns the evaluation of the models in order to demonstrate their reliability and accuracy in predicting mortality in critical care patients.

### 3.1. Datasets

This study was based on three public critical care databases provided by the MIT Laboratory for Computed Physiology:

- **MIMIC-III** [11] (v1.4, released in 2016) is a critical care database of over 46,000 de-identified patients with over 60,000 admissions obtained from the Beth Israel Deaconess Medical Center (BIDMC) between 2001 and 2012. The database contains demographics, diagnoses, vital signs, laboratory tests, doctor notes, etc.
- **MIMIC-IV** [12] (v0.4, released in 2020) is a critical care database of more than 200,000 emergency department stays and 60,000 intensive care stays obtained from the BIDMC between 2008 and 2019. The database also contains the relevant clinical data prior to ICU admission.
- **eICU** [13] (v2.0, released in 2018) is a critical care database of more than 200,000 intensive care stays collected between 2014 and 2015 from hospitals in the United States that participate in the Philips eICU Program. The database contains demographics, diagnoses, treatment information, care plan documents, vital signs, laboratory tests, doctor notes, etc.

**Figure 1:**
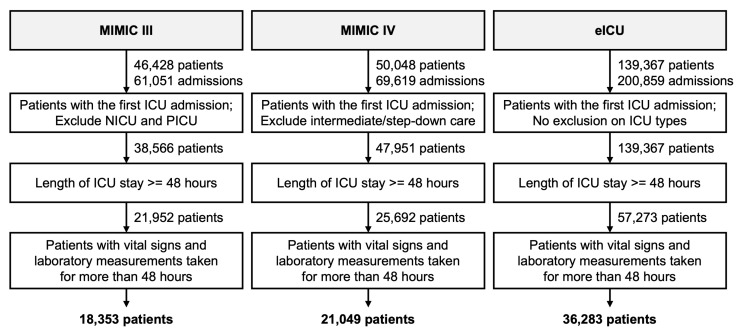
Flow chart for study cohort formation.

Similar cohort selection criteria were applied for all databases (see Figure 1). We included only the first admission of each patient in the ICU whose length of stay in the ICU was longer than 48 h. This resulted in each patient having only one ICU admission. For MIMIC-III, patients admitted to the neonatal intensive care unit (NICU) and pediatric intensive care unit (PICU) were excluded. For MIMIC-IV, patients admitted to intermediate or step-down care units were excluded. We further excluded patients for whom the duration between the first and last observations of vital signs and laboratory tests was less than 48 h (e.g., a patient with a record of 53 h in MICU but whose first measurements were not made until after hour 6 was excluded). The duration was calculated as the last timestamp minus the first timestamp in the chartevents/labevents table.

Patients who died during their ICU stay were identified by the deathtime variable in the admission table of MIMIC-III, the hospital_expire_flag variable in the admission table of MIMIC-IV, and the unitdischargestatus variable in the patients table of eICU. Otherwise, patients were assumed to have survived until the ICU discharge. Table 1 summarizes the patient demographics of our study cohorts for each database.

### 3.2. Data Exploration

The MIMIC-III/IV and eICU databases contain a large number of clinical variables. In consultation with critical care experts, only vital signs and laboratory variables available in conventional clinical scoring systems and in all databases were included in our study. Each measurement in the critical care databases was associated with a timestamp and variable name as defined in the original electronic health record (EHR). Thus, the same clinical variable could be associated with different names and units. The database scripts provided in the official repository of each critical care database were utilized to handle outliers and semantically group similar clinical variables. Table 2 summarizes the clinical variables used to develop our prediction algorithms. Of these, 7 were vital sign variables, and 16 were laboratory variables.

### 3.3. Data Preparation

Vital sign measurements were typically taken 0.5–1.5 times per hour for the MIMIC-III and MIMIC-IV databases and 2.5–20 times per hour for the eICU database, while laboratory measurements were typically taken 1–2 times per eight hours for all databases [29]. Therefore, each vital sign variable was aggregated into a one-hour interval, whereas each laboratory variable was aggregated into an eight-hour interval. Repeated measurements in a single interval were aggregated by the median. For example, if five measurements were taken in the fifth hour after admission, these measurements were aggregated horizontally into one value by using the median to represent the value for that hour interval. We observed no differences in results when we used the mean or median for data aggregation. Missing measurements were imputed by carry-forward imputation (i.e., the last measurement is carried forward if the current measurement was not available). The values of the variables that were never observed were set to −1.

The common approach for developing ML algorithms is to divide the data into two parts: one for hyperparameter tuning and one for testing the developed model. For each critical care database, 15% of the dataset was kept as a test set, while the other 85% was used to develop algorithms through a cross-validation scheme with five independent folds (k=5), similar to El-Rashidy et al. [16]. The resulting models trained on k−1 folds and validated on the kth fold were then evaluated on the hold-out test set.

### 3.4. Recurrent Neural Networks

RNN is a feed-forward neural network in which the output of the previous step is fed as input to the current step as prior information. With its sequential mechanism, RNN is capable of processing sequences of variable length inputs. RNNs are usually implemented with additional stored states with controlled time delays or feedback loops, allowing the learning of both short-term and long-term temporal dynamics. In this work, we applied RNN to the task of time-series classification in which a patient’s time-series data were fed into the network and the output indicating whether the patient survived or passed away was then produced. We further incorporated the explainability module to provide the causal explanation behind the decision made by the network.

#### 3.4.1. Problem Formulation

**Figure 2:**
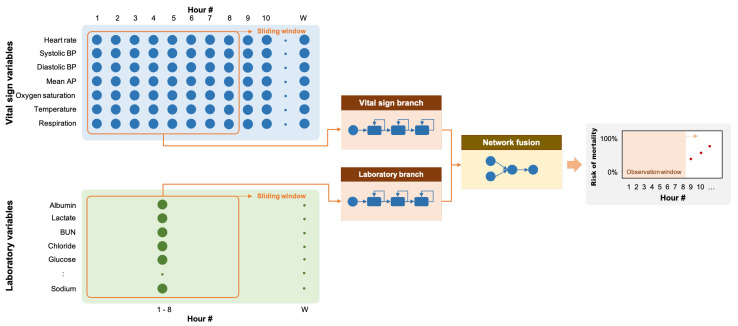
Diagram of our RNN model with two input branches for vital signs and laboratory variables, respectively. Each branch consists of three recurrent layers, and the resulting feature maps were fused with two fully connected layers. The network ends with a sigmoid layer for estimating a risk of mortality from 0 to 1.

ICU mortality is defined as death while the patient is in the ICU. The problem of predicting ICU mortality was formulated as a multivariate binary time-series classification problem in which patients who died during their stay in the ICU were included in the positive group (output = 1), and patients who survived to discharge were included in the negative group (output = 0). The time points indicating the mortality event was defined as the last time point at which vital signs or laboratory measurements were taken or the deathtime variable for the positive group in the eICU database. $T_{O}$ hours of vital signs (7 variables) and laboratory observations (16 variables) before this time point were taken as the observation window. The data from this window were extracted and used for training and evaluation of the predictive models. The resulting algorithm was then able to indicate the likelihood or probability that a patient would have mortality at the end of the time window (see Figure 2). For the negative group, the observation window was defined as $T_{O}$ hours of vital signs and laboratory observations before patient discharge.

#### 3.4.2. Network Architecture

**Figure 3:**
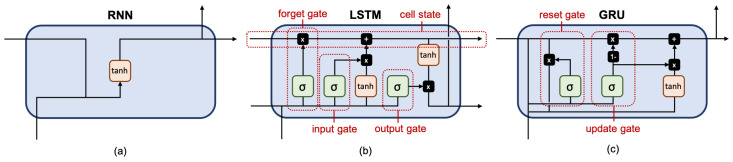
Schematic diagram of each recurrent unit with different stored states: (**a**) FRNN with a hidden state, whose activation at each time depends on that of the previous time; (**b**) LSTM has a cell state and multiple control gates to modulate the flow of information so that each recurrent unit can adaptively perceive dependencies on different time scales; (**c**) GRU simplifies the input gate and forget gate of LSTM into an update gate.

Since we have two sets of clinical variables with different temporal characteristics, we implemented our RNN model with two input branches: one for the vital sign variables and the other for the laboratory variables. Each branch was implemented with three recurrent layers with 16 units each, followed by batch normalization. The outputs of the two recurrent branches were then concatenated along the channel dimension. The resulting feature maps were processed through two fully-connected layers with eight outputs and one output, respectively. The network ended with a sigmoid layer to produce a predicted risk between 0 and 1. We evaluated three different recurrent layers:

- **The fully-connected RNN (FRNN)** [30] (see Figure 3a) connects the output of the previous time step with the additional input of the next time step, preserving important information about different time steps in the network.
- **The long short-term memory (LSTM)** [31] (see Figure 3b) has one cell state and three gates: an input gate, an output gate, and a forget gate. The cell state acts as a memory, while each gate functions like a conventional neuron, providing a weighted sum of its inputs. The forget gate decides what information to retain from previous steps. The input gate decides what information to add from the current step. The output gate decides what the next hidden state should be. Hence, only relevant information can pass through the hierarchy of the network. Thus, the LSTM has mechanisms to process both short-term and long-term memory components.
- **The gated recurrent unit (GRU)** [32] (see Figure 3c) is similar to LSTM but has only two gates: an update gate and a reset gate. The update gate works similarly to the forget gate and the input gate of LSTM. It decides what information to throw away and what to add. The reset gate decides how much past information to forget. GRU has fewer parameters, uses less memory, and is faster to train than LSTM.

The name of a recurrent layer was taken as the name of the model. Our baseline model was implemented using a feed-forward multilayer perceptron (MLP) [30] with three fully-connected layers each with 64 neurons. MLP took all values in the time window as the input.

The number of layers and the number of neurons given above were obtained through a heuristic search over different combinations of hyperparameters (i.e., the number of layers l={1,2,3,4} and the number of neurons n={8,16,32,64,128}). We selected a set of hyperparameters in which the evaluation metric (AUC) seems saturated across different cross-validation folds with a standard deviation of less than 0.05.

#### 3.4.3. Network Optimization

Our models were optimized using Adam [33] with a binary cross-entropy loss, a batch size of 64, and an initial learning rate of 0.01 (0.001 for the eICU database) and reduced by half every five epochs until convergence. For the positive group, oversampling was performed at random to match the number of samples with those of the negative group. Each training batch, therefore, generally has an equal number of samples for each class. Each branch was first trained separately and later combined into a single model, followed by retraining the entire model.

#### 3.4.4. Explainability Module

A fundamental question we might be asked is: which features have the greatest influence on predictions? SHAP (SHapley Additive exPlanations) [25] is an additive feature attribution method that explains model prediction through the allocation of the values among a set of input features. In SHAP, the impact on each feature is represented by Shapley values and is defined as the change in the expected value of the model output when a feature is present versus unknown. Some features may have a large impact, while others may have a small impact on the prediction.

Given a prediction f(x) made with all *N* input features, Shapley values can be calculated as a weighted average of the contribution of each feature *i* over all possible feature combinations:

(1) $$\phi_{i}\left(f,x\right)=\sum_{S\subseteq S_{all}\setminus \left\{i\right\}}\frac{|S|!(N-|S|-1)!}{N!}\left[f_{x}\left(S\cup \left\{i\right\}\right)-f_{x}\left(S\right)\right]$$

where *S* denotes a subset of the features used in the model, excluding the *i*-th feature. To calculate the exact Shapley value for the *i*-th feature, we have to evaluate all possible feature combinations with and without the *i*-th feature. In practice, it is not possible to evaluate all features, but we can approximate these values by a sampling procedure (e.g., Monte-Carlo sampling). For deep learning, SHAP assumes that the input features are independent and that the deep learning model is linear. SHAP values can be approximated through a simpler explanation model learned from the original model.

In our work, SHAP values were computed for the models constructed from each cross-validation fold using data from the test set. Average SHAP values were then calculated for each feature and for each time step, indicating the impact of that feature or time step on the model’s output.

#### 3.4.5. Tools

Cohort selection and data preparation were performed using SQL operations on the PostgreSQL database server (version 10). Our models were developed using Python version 3.8.5, TensorFlow version 2.2.0, and SHAP version 0.38.0 with custom modifications to allow interpreting RNN models with multiple time-series inputs. All experiments were performed on a workstation with 8 processing cores and 32GB RAM.

### 3.5. Evaluation

Evaluation was performed using the data from the hold-out test set on the model that was trained and validated from each cross-validation fold. Given that a true positive (TP) is a result of the model correctly predicting mortality (the positive group), a true negative (TN) is a result of the model correctly predicting survival to discharge (the negative group), a false positive (FP) is a result of the model incorrectly predicting the positive group, and a false negative (FN) is a result of the model incorrectly predicting the negative group. Our models were evaluated through the following metrics commonly used for binary classification:

(2) $$\mathrm{Accuracy}=\frac{TP+TN}{TP+TN+FP+FN}$$

(3) $$\mathrm{Sensitivity}=\frac{TP}{TP+FN}$$

(4) $$\mathrm{Specificity}=\frac{TN}{TN+FP}$$

(5) $$\mathrm{Positive}\;\mathrm{Predictive}\;\mathrm{Value}\;(\mathrm{PPV})=\frac{TP}{TP+FP}$$

(6) $$\mathrm{Negative}\;\mathrm{Predictive}\;\mathrm{Value}\;(\mathrm{NPV})=\frac{TN}{TN+FN}$$

(7) $$F_{1}\;\mathrm{Score}=\frac{2TP}{2TP+FP+FN}$$

A receiver operating characteristic curve (ROC) is generated by plotting sensitivity and 1-specificity values at different classification thresholds. The area under the ROC (AUC) was used as a single performance metric describing the overall classification performance of the model. Youden’s J statistic was used to compute the optimal classification threshold from the ROC curve for each model trained from each cross-validation fold. The AUC is a standard metric for reporting binary classification algorithms derived from PhysioNet studies so that different studies can be compared across the research landscape. The mean and standard deviation of each evaluation metric was then computed from the results obtained from the different cross-validation folds.

## 4. Results

In this section, we detail the results obtained from (1) different RNN models with a 48-h observation window (T${}_{O}$ = 48), (2) the best-performing RNN models with 8/16/24/48-h observation windows (T${}_{O}$ = 8/16/24/48), and (3) the feature importance derived form the best-performing RNN model.

### 4.1. Prediction of Mortality

**Figure 4:**
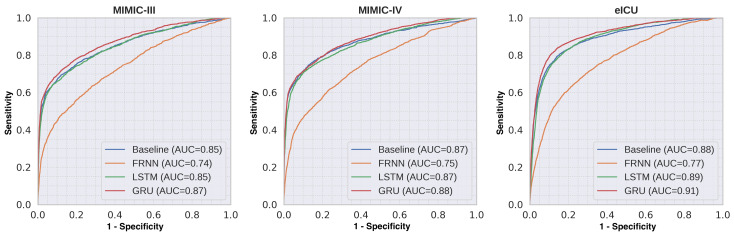
ROC curves comparing different RNN models trained on all variables for each database with the 48-h observation window. The GRU models had the highest AUCs. Similar trends were observed across different databases.

We evaluated the classification performances of each model (baseline MLP, FRNN, LSTM, and GRU) on three critical care databases. The evaluation was performed on the hold-out test set of each database. Table 3 shows the comparative performance of the different RNN models for predicting mortality with a 48-h observation window (T${}_{O}$ = 48) based on their AUCs. The GRU model trained with all clinical variables had the highest AUCs of 0.87, 0.88, and 0.91 for the MIMIC-III, MIMIC-IV, and eICU databases, respectively. For the models trained with laboratory variables only, we observed much lower classification performance for all databases. Similar trends were observed for all databases. Figure 4 shows the mean ROC curves of the different RNN models trained with all variables. Table 4 shows the detailed classification performance measures of the best-performing GRU model. Youden’s J statistic was applied to find an optimal threshold from the ROC curves in order to compute classification metrics. Sensitivity and specificity can be varied by changing the threshold level.

Table 5 compares the performance of the GRU models with different observation window lengths. The model trained with an 8-h observation window (T${}_{O}$ = 8) showed slightly lower discrimination performance compared to the models trained with larger observation windows.

### 4.2. Feature Importance

**Figure 5:**
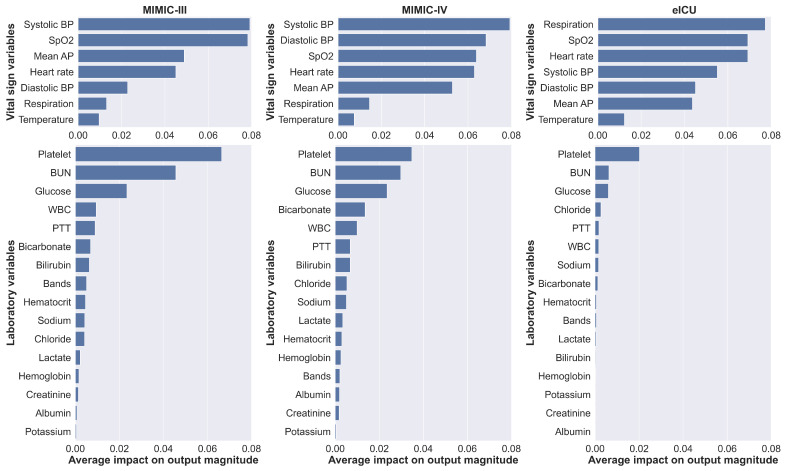
Feature importance indicating each feature’s average impact on the model prediction, ranked by the degree of importance for both vital sign and laboratory variables. Reasonable agreement was observed across databases.

SHAP was used to interpret feature importance by calculating the contribution of each feature to model output. We averaged the values of SHAP separately over all variables and time steps to obtain a general understanding of each feature’s impact on prediction. Figure 5 shows the ranking of feature importance for both vital sign and laboratory variables. In general, reasonable agreement of feature importance was observed across different databases.

For the vital sign variables, three of the top four important features (systolic BP, SpO2, and heart rate) were shared for the MIMIC-III and MIMIC-IV databases. We found that respiratory rate was the strongest feature in the eICU database, in contrast to the MIMIC-III and MIMIC-IV databases, where respiratory rate was ranked second to last. Note that the MIMIC-III and MIMIC-IV databases were derived from the same hospital, whereas the eICU database was derived from multiple hospitals. In addition, for the eICU database, vital signs were available more frequently, whereas in the MIMIC-III and MIMIC-IV databases, measurements were available hourly.

For the laboratory variables, the three most important features (platelet count, BUN, and glucose) were the same in all databases. Conversely, the three least important features (creatinine, albumin, and potassium) were also similar. We found that the models gave more importance to the vital sign variables than to the laboratory variables. This trend was supported by the previous section that the models trained with only vital sign variables had higher discriminatory power than the models trained with only laboratory variables. This trend was more prominent for the eICU database.

**Figure 6:**
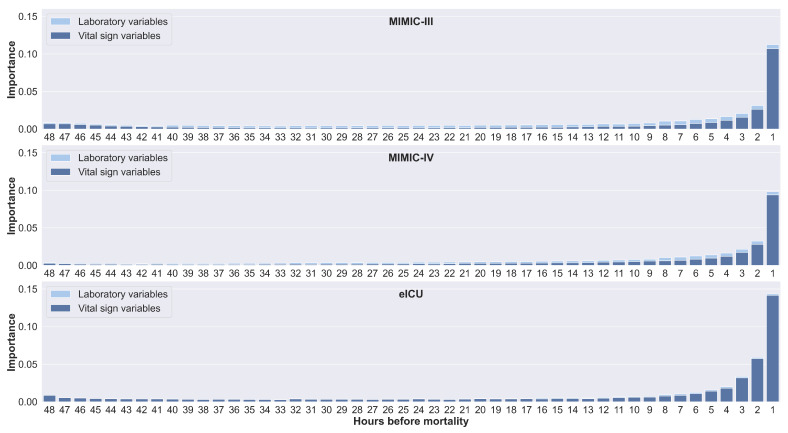
Feature importance for each time step, calculated based on the 48-h window prior to mortality. Values from the time steps preceding the mortality event have a large impact on the prediction. The values of vital signs from the earliest time steps were also observed to impact the prediction.

Figure 6 shows the feature importance calculated for each time step in the 48-h time window by horizontally stacking all feature importance values for each time step. Similar trends were observed for all databases. Values from the time steps preceding the mortality event had a large impact on the prediction. In addition, values of vital signs from the earliest time steps also have an impact on prediction. This could explain why only a slight decrease in performance was observed in Table 5 for an 8-h window compared to a 48-h window.

## 5. Discussion

This section discusses our results, compares our work with other literature, gives a more complete picture of our proposed methods, and provides the limitations of our study.

### 5.1. Prediction of ICU Mortality

Traditional early warning score scoring systems, such as APACHE II and MEWS, evaluate only the most recently collected vital signs and assume that each variable is independent of the other variables. We show that historical observations can provide potentially useful additional information for the development of automatic scoring algorithms. We found that FRNN achieved low prediction scores, which may be caused by gradient vanishing, which is a common problem of FRNN that makes it unsuitable for processing long sequences. LSTM and GRU, on the other hand, have dedicated processing gates that learn what data are most important for the prediction. This allows the network to learn important and relevant features and maintain them regardless of the state. For the GRU models, we observed a 1%–5% increase in AUC over the baseline models.

For the eICU database, we also found that the model tends to favor vital signs over laboratory observations. This could be due to the fact that multiple vital sign measurements are aggregated into hourly values, resulting in less variation in values.

### 5.2. Comparison across Different ICU Types

ICUs are usually divided into different types, each designed to meet the specific needs of patients. Trauma ICUs, for example, are staffed with a dedicated team that specializes in treating patients with severe trauma. Algorithms trained with clinical data obtained only from patients treated in a single ICU may show different performance when evaluated with clinical data from patients treated in other ICUs due to differences in disease characteristics.

Table 6 shows the performances of the GRU model trained with data from patients treated in all ICUs in the 48-h window for each database and for each ICU type. We observed similar performances for the AUCs of the different ICU types. A slight decrease in performance is expected for the surgical ICU, where a portion of the patients is in the postoperative recovery phase.

### 5.3. Feature Importance

SHAP was employed to gain insight into general factors that impact the prediction. Figure 5 shows each group of features sorted by the magnitude of their importance. The prediction was mainly influenced by a few features. Among the vital sign features, the MIMIC-III/IV and eICU databases reported different sets of feature importance, with the respiratory rate ranked as the most impact feature in the eICU database. In the MIMIC-III/IV databases, the respiratory rate was checked hourly at the bedside with verification by nursing staff. In contrast, in the eICU database, vital signs were automatically derived from bedside vital sign monitors, without further verification for up to 20 measurements per hour; therefore, measurements may be noisy [13]. We preprocessed the data by taking the median across multiple measurements at an hourly interval. Cretikos et al. [34] suggested that an accurate respiratory rate could be an important predictor of serious clinical events. We hypothesized that although the respiratory rate is considered noisy and difficult to measure by contact sensors, the periodically recorded values, if properly processed and aggregated over multiple measurements, might better reflect the true condition of patients.

For the laboratory features, Moreno et al. [6] examined several clinical variables for the development of a prognostic model for ICU mortality using logistic regression in 16,000 patients. The authors reported that the platelet count had the highest laboratory coefficient in relation to the outcome. This was supported by Moreau et al. [35], whose study reported that decreasing platelet counts were associated with higher mortality rates; therefore, it was suggested that the platelet count should be included in a scoring system. In addition, it has been reported that BUN, the second most important feature, is strongly associated with mortality, with a high BUN on admission to the ICU being considered an independent risk factor for patient mortality [36]. In terms of blood glucose, hyperglycemia, even if it is only a mild elevation in blood glucose, has been reported to be common in critically ill patients, regardless of diabetes [37].

For the features with the least impact, Gall et al. [5] observed that albumin and creatinine had no significant effect on ICU mortality in their univariate and bivariate analyses and did not improve goodness of fit. This is consistent with our findings on feature importance.

### 5.4. Explainability

SHAP provides the explainability of the prediction by calculating the contribution of each feature to the model output. These explanations can be coded in a form that allows clinical staff to easily interpret, in both variable and time dimensions, why particular decisions were made.

**Figure 7:**
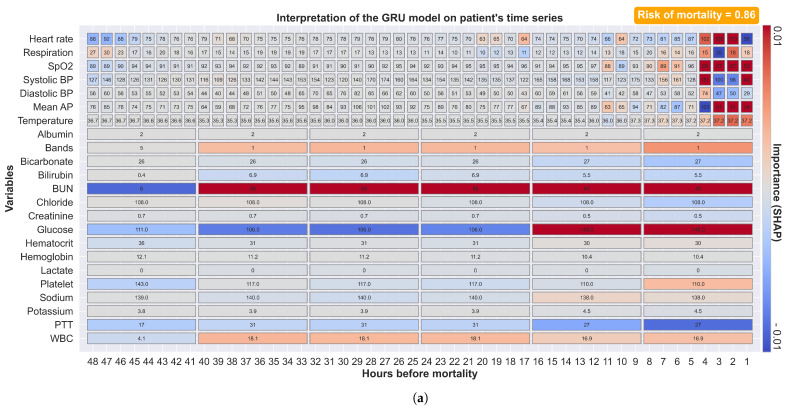

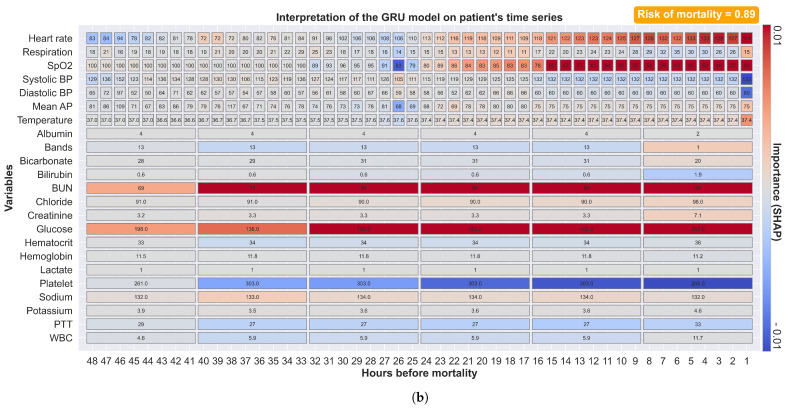
Visualization of SHAP values calculated by our GRU model for 48 h of time-series data preceding mortality. The values of each clinical variable at each time step are presented in each rectangular box, with missing observations filled using carry-forward imputation. The color red indicates positive SHAP values (increased mortality risk), while the color blue indicates negative SHAP values (decreased mortality risk). (**a**) Our approach was able to recognize changes in both vital signs (four hours before mortality for heart rate, SpO${}_{2}$, and blood pressure) and laboratory tests (forty hours before mortality for bilirubin, BUN, PTT, and WBC). (**b**) The prediction based on data with a longer period of vital sign alterations. Our model gives more weight to more recent measurements. Our model was able to adapt to specific patients.

Figure 7a shows a prediction made 48 h before mortality with the corresponding explanation. The color red indicates positive SHAP values (increased risk of mortality), while the color blue indicates negative SHAP values (decreased risk of mortality). Vital sign alterations were observed four hours before mortality with an increased heart rate and decreased SpO${}_{2}$ and blood pressure. At forty hours before mortality, an increase in bilirubin, BUN, PTT, and WBC was observed. At sixteen hours before mortality, an increase in glucose was observed. Our model explains that a mortality probability of 0.86 was constituted from these observations; each with different weighting factors. Our approach provides not only the predicted mortality risk but also information about the factors that influence the risk and their relative contributions. This explanatory diagram helps clinical staff to quickly identify apparent relationships between patient physiology and trends in their contribution to the risk over time.

Figure 7b shows another example of a prediction where mortality is preceded by a longer period of physiological alterations. Our model weights more recent measurements more heavily. Our model also identified a large increase in creatinine, although this was ranked as one of the least important features. These explanatory features illustrate not only the ability of our complex models to dynamically explain predictions but also provide general insights into changes in specific patients. This augmented-intelligence approach provides clinical staff with a more clinically useful interpretation. On the contrary, our model outputs all available observations with their associated impacts, and clinical staff must evaluate their relevance based on context and other clinical evidence.

### 5.5. Risk of Mortality

**Figure 8:**
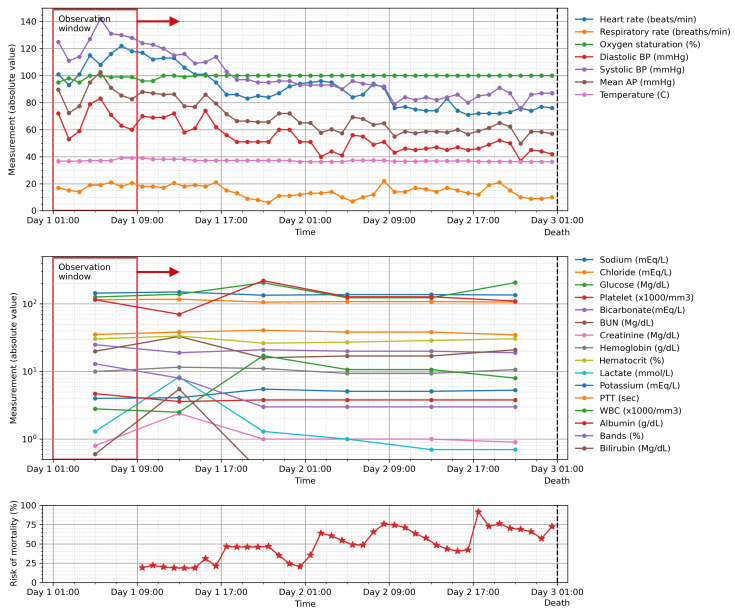
The risk of mortality calculated for a patient who later died during their ICU stay in the hold-out test set computed using the GRU model with an 8-h observation window. The values increased continuously as the patient’s clinical condition worsened until the patient passed away at the end of their stay.

Our model provides a probability value for mortality at the end of the observation period, as derived from the last sigmoid layer of the model whose output ranges from 0 to 1. The value could be used to indicate the deterioration of the patient’s condition in order to track and trigger an escalated care plan if necessary. Figure 8 shows the calculation of such value for a sliding window of 8 h for a patient who died during his stay in the ICU. The risk of mortality increased continuously as the patient’s clinical condition worsened until the patient passed away at the end of their ICU stay.

**Figure 9:**
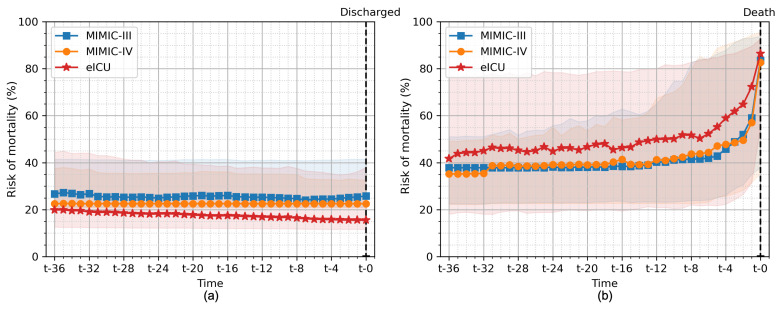
The risk of mortality calculated for all patients in the hold-out test set for (**a**) those patients who survived their ICU stay and (**b**) those patients who subsequently died during their ICU stay. The line plots show the median, and the shaded areas show the 25th and 75th quartiles of risk of mortality bootstrapped from all cross-validation folds. Among the surviving patients, the risk of mortality was low and tended to decrease until discharge. In contrast, among patients who died during their stay, the risk of mortality was high and increased until the patient’s death. A sharp increase in the risk of mortality was observed eight hours before death.

Figure 9 shows the average of risk of mortality for all patients in the hold-out test set evaluated on all the different cross-validation folds for patients who survived their ICU stay and patients who subsequently died during their ICU stay. We used our GRU model with an 8-h observation window (the same model as in Figure 8). For surviving patients, we observed a slight decrease in the risk of mortality until the patient was discharged from the ICU. In contrast, the risk of mortality was high in patients who died during their stay and increased until the patient’s death. The value of more than 50% was noted 8 h and 4 h before death in the eICU and MIMIC-III/IV databases, respectively.

These types of automated scoring systems, which process both vital signs and laboratory observations routinely collected in the HIS, could be integrated into bedside monitoring or into a dashboard for monitoring the entire ICU. The explainability of the model could highlight important insights for further interpretation by clinical staff. With continuous monitoring in real time, patients can benefit from early interventions.

### 5.6. Comparison with Other Studies

Our results are consistent with other relevant studies. Here, we compared our results with studies based on the MIMIC-III database. Our best-performing model has slightly lower performance in comparison to similar studies by Johnson et al. [15] (0.87 vs. 0.92), El-Rashidy et al. [16] (0.87 vs. 0.93), and Purushotham et al. [19] (0.87 vs. 0.94). In contrast to our study, Johnson et al. [15] included other clinical variables, such as the Glasgow coma components, and clinical details (e.g., gender, age, type of ICU, etc.) in their model. El-Rashidy et al. [16] used over 80 clinical parameters and the ensemble technique over multiple classifiers. Purushotham et al. [19] used over 135 clinical features with multiple deep learning models. Nevertheless, our study used only 23 clinical variables that are clinically relevant to mortality. We found that the more clinical variables included in the model, the better the results.

In RNN-based studies, our model achieved on par performance compared to other mortality prediction studies by Harutyunyan et al. [22] (0.87 vs. 0.87) and Jun et al. [21] (0.87 vs. 0.87). Harutyunyan et al. [22] used a multi-task LSTM to predict multiple clinical events, including mortality, with 17 clinical variables. Jun et al. [21] used a variational RNN with 99 clinical variables. All of the aforementioned studies were conducted using the MIMIC-III database. Similar to our study, Shamout et al. [24] developed an interpretable bidirectional LSTM to predict clinical adverse events, including mortality, in the next 24 h based on their institutional dataset. They reported an AUC of 0.88 for predicting adverse events. We did not perform a direct comparison with conventional EWSs. Such comparisons were made in the studies of Purushotham et al. [19], Harutyunyan et al. [22], and Shamout et al. [24]. All the studies yielded higher AUCs than those of the conventional EWSs.

### 5.7. Limitations

Our study was subject to several limitations. First, we compared the performance of only the conventional approaches. Improvements could be achieved by integrating more complex mechanisms, such as bidirectional LSTM or attention-based LSTM. Second, we observed a class imbalance where about 8%–16% of the patients in each database belonged to the positive group. We treated this with simple over-sampling before training. A more complicated balancing method could lead to improving the model. Finally, we observed slight differences in the feature importance values for the models trained on the eICU database compared to the models trained on the MIMIC databases. This could be due to the fact that the data have different intrinsic characteristics. This could indicate that the model trained on one database may not generalize well to other databases or other environments. From a deployment perspective, the generalizability of the proposed model needs to be further tested on a locally acquired dataset or transfer learning to a local dataset may be required.

## 6. Conclusions

The ICU generally cares for critically ill patients who require life-sustaining measures and specialized treatment. Early prediction of mortality in the ICU is crucial to identify patients who are at high risk of death and take appropriate interventions to save their lives. Although various severity scores and ML models have been developed recently for the early prediction of mortality, this prediction remains a challenge. This study proposes a data-driven framework for predicting mortality risk of mortality in intensive care patients using the multiple-input RNN architecture coupled with the explainability module. Our algorithm processes time-series of vital signs and laboratory results and produces a predicted mortality risk score along with the contribution of each input feature to the prediction. We evaluated our approach using the same pipeline on three recent critical care databases: MIMIC-III, MIMIC-IV, and eICU. Consistent results were obtained on the different critical care databases, demonstrating the robustness of our approach. Our proposed method yielded an AUC of 0.87, 0.88, and 0.91 for MIMIC-III, MIMIC-IV, and eICU, respectively. The prediction made by our model was consistent with clinical events (survival to ICU discharge and ICU mortality). Through SHAP, our approach was able to explain the factors associated with mortality for each individual patient in both variable and time dimensions, avoiding the obscurity associated with complex black-box models. The explanation and feature importance derived from our model were consistent with the literature. We coded these explanations in a visual form that clinical staff can easily interpret. Patients may benefit from early interventions with continuous monitoring of their clinical measurements in real time. Future work includes the transfer learning of the proposed algorithm to our institutional critical care data and the investigation of different architectures, such as bidirectional transformers for multi-step ahead prediction.

## Figures and Tables

**Table 1:** Demographics for our study cohorts derived from the MIMIC-III, MIMIC-IV, and eICU databases.

Demographics	MIMIC-III			MIMIC-IV			eICU		
	Total	Survival ${}^{1}$	Death ${}^{2}$	Total	Survival ${}^{1}$	Death ${}^{2}$	Total	Survival ${}^{1}$	Death ${}^{2}$
Number of patients	18,353	15,761	2592	21,049	18,134	2915	36,283	33,387	2896
Age (years) ${}^{3}$	65.4 (17.9)	64.5 (17.9)	71.0 (17.0)	65.0 (16.6)	64.8 (16.7)	69.7 (15.4)	65.0 (17.1)	64.8 (17.2)	67.3 (16.3)
ICU stay (days) ${}^{3,4}$	6.1 (7.4)	5.9 (7.1)	8.1 (8.3)	5.9 (6.7)	5.6 (6.4)	8.0 (8.0)	5.1 (5.0)	5.0 (4.7)	7.2 (7.6)
Gender									
Male	10,221	8838	1383	11,854	10,267	1587	19,945	18,334	1611
Female	8132	6923	1209	9195	7867	1328	16,333	15,049	1284
Unknown	−	−	−	−	−	−	5	4	1
Ethnicity									
Caucasian	12,976	11,232	1744	14,028	12,291	1737	28,542	26,228	2314
African American	1376	1232	144	1784	1574	210	4075	3774	301
Hispanic/Latino	559	511	48	699	628	71	1420	1332	88
Asian	424	373	51	567	486	81	444	399	45
Others/Unknown	3018	2413	605	3971	3155	816	1802	1654	148

^1^ Survival to ICU discharge; ^2^ Death in ICU; ^3^ Values shown in mean (standard deviation); ^4^ Length of the first ICU stay.

**Table 2:** The distribution of the values of each variable employed in our models. All variables are present in every dataset.

Clinical Variables	Valid Range		MIMIC-III		MIMIC-IV		eICU	
	Lower	Upper	Survival ${}^{1}$	Death ${}^{2}$	Survival ${}^{1}$	Death ${}^{2}$	Survival ${}^{1}$	Death ${}^{2}$
**Vital sign variables** (7 variables)								
Heart rate (beats/min)	0	350	84.3 (16.1)	90.4 (19.8)	84.4 (16.7)	91.3 (19.8)	84.2 (17.1)	93.1 (20.8)
Diastolic blood pressure (mmHg)	0	375	61.4 (14.2)	56.8 (14.4)	63.3 (14.6)	57.5 (14.3)	67.9 (14.4)	60.9 (15.1)
Systolic blood pressure (mmHg)	0	375	124.5 (21.4)	116.4 (25.6)	122.6 (21.0)	113.5 (24.5)	126.1 (22.1)	115.4 (24.7)
Mean arterial pressure (mmHg)	14	330	80.5 (15.3)	75.4 (17.1)	79.3 (15.3)	73.8 (16.7)	83.9 (16.1)	76.0 (16.9)
Temperature (${}^{\circ }$C)	26	45	36.9 (0.6)	36.9 (1.0)	36.9 (0.5)	36.9 (0.8)	36.8 (0.5)	36.8 (1.1)
Peripheral oxygen saturation (%)	0	100	96.8 (2.6)	95.6 (6.4)	96.3 (2.6)	95.3 (6.1)	96.3 (3.2)	95.2 (7.2)
Respiratory rate (breaths/min)	0	300	19.9 (5.4)	21.4 (6.7)	19.7 (5.4)	21.4 (6.4)	19.5 (5.2)	21.9 (7.2)
**Laboratory variables** (16 variables)								
Albumin (g/dL)	0.6	6	2.6 (0.5)	2.5 (0.6)	2.8 (0.5)	2.7 (0.6)	2.6 (0.6)	2.4 (0.6)
Blood urea nitrogen (mg/dL)	0	250	36.9 (26.5)	50.4 (31.8)	38.0 (26.8)	44.6 (32.4)	26.3 (21.2)	39.8 (28.2)
Bilirubin (mg/dL)	0.1	60	3.6 (5.3)	10.5 (13.1)	3.9 (6.4)	7.9 (10.1)	1.4 (3.2)	3.2 (6.1)
Lactate (mmol/L)	0.4	30	1.9 (1.5)	3.8 (4.2)	1.9 (1.4)	3.5 (3.4)	2.1 (2.0)	5.5 (5.0)
Bicarbonate (mEq/L)	0	60	25.8 (4.7)	23.9 (5.7)	25.8 (5.3)	23.0 (5.7)	25.6 (4.9)	22.3 (6.0)
Band neutrophil (%)	0	100	5.2 (6.3)	6.4 (7.2)	4.6 (5.7)	5.2 (5.6)	8.6 (12.6)	12.2 (13.1)
Chloride (mEq/L)	50	175	105.3 (6.1)	103.9 (7.3)	103.6 (7.2)	103.3 (7.9)	104.3 (6.7)	106.8 (9.2)
Creatinine (mg/dL)	0.1	60	1.4 (1.4)	1.8 (1.3)	1.6 (1.5)	1.8 (1.3)	1.3 (1.4)	2.0 (1.6)
Glucose (mg/dL)	33	2000	131.6 (52.2)	136.9 (64.3)	140.6 (62.3)	147.8 (67.4)	144.2 (57.2)	149.9 (62.9)
Hemoglobin (g/dL)	0	25	9.6 (1.4)	9.6 (1.4)	9.1 (1.6)	8.9 (1.4)	10.2 (2.0)	9.8 (2.1)
Hematocrit (g/dL)	0	75	28.6 (3.9)	28.6 (3.9)	27.8 (4.6)	27.2 (4.3)	31.1 (6.1)	29.8 (6.5)
Platelet count (1000/mm${}^{3}$)	0	2000	278.9 (192.2)	162.5 (138.9)	237.0 (174.2)	154.3 (134.8)	208.3 (115.7)	153.4 (105.5)
Potassium (mEq/L)	0	12	4.0 (0.5)	4.1 (0.6)	4.0 (0.5)	4.1 (0.6)	3.9 (0.5)	4.2 (0.8)
Partial thromboplastin time (s)	18.8	150	44.7 (23.7)	52.9 (28.0)	47.7 (24.4)	52.9 (26.7)	50.7 (27.7)	52.2 (27.9)
Sodium (mEq/L)	50	225	140.3 (5.2)	139.1 (6.0)	140.4 (5.9)	139.5 (6.7)	138.9 (5.7)	141.7 (8.1)
White blood cells (1000/mm${}^{3}$)	0	1000	12.9 (8.5)	14.7 (9.8)	12.9 (8.9)	15.7 (13.7)	11.2 (6.0)	15.8 (9.7)

^1^ Survival to ICU discharge; ^2^ Death in ICU; Values shown in mean (standard deviation).

**Table 3:** Performance of different RNN models with a 48-h observation window (T${}_{O}$ = 48).

Variables	Dataset	AUC (Mean ± Standard Deviation)			
		MLP	FRNN	LSTM	GRU
Vital signs	MIMIC-III	0.81 ± 0.02	0.68 ± 0.05	0.83 ± 0.01	**0.85 ± 0.01**
	MIMIC-IV	0.83 ± 0.02	0.70 ± 0.02	0.86 ± 0.01	**0.87 ± 0.01**
	eICU	0.86 ± 0.01	0.74 ± 0.03	**0.89 ± 0.01**	**0.89 ± 0.01**
Laboratory variables	MIMIC-III	0.67 ± 0.02	0.66 ± 0.03	0.68 ± 0.01	**0.69 ± 0.01**
	MIMIC-IV	0.65 ± 0.03	0.63 ± 0.03	**0.66 ± 0.01**	**0.66 ± 0.02**
	eICU	0.59 ± 0.02	0.55 ± 0.02	**0.61 ± 0.02**	**0.61 ± 0.03**
All variables	MIMIC-III	0.85 ± 0.01	0.74 ± 0.01	0.85 ± 0.01	**0.87 ± 0.01**
	MIMIC-IV	0.87 ± 0.01	0.75 ± 0.04	0.87 ± 0.01	**0.88 ± 0.01**
	eICU	0.88 ± 0.01	0.77 ± 0.03	0.89 ± 0.02	**0.91 ± 0.01**

**Table 4:** Classification performance measures of the GRU model with a 48-h observation window (T${}_{O}$ = 48).

Variables	Dataset	Classification Metrics (Mean ± Standard Deviation)						
		AUC	Accuracy	Sensitivity	Specificity	PPV	NPV	F${}_{1}$ Score
Vital signs	MIMIC-III	0.85 ± 0.01	0.78 ± 0.01	0.63 ± 0.03	0.91 ± 0.01	0.88 ± 0.02	0.71 ± 0.01	0.74 ± 0.01
	MIMIC-IV	0.87 ± 0.01	0.81 ± 0.01	0.71 ± 0.04	0.90 ± 0.03	0.88 ± 0.03	0.76 ± 0.02	0.79 ± 0.02
	eICU	0.89 ± 0.02	0.82 ± 0.01	0.78 ± 0.03	0.86 ± 0.03	0.84 ± 0.02	0.81 ± 0.01	0.82 ± 0.01
Laboratory variables	MIMIC-III	0.69 ± 0.02	0.64 ± 0.01	0.56 ± 0.06	0.71 ± 0.07	0.66 ± 0.04	0.62 ± 0.01	0.60 ± 0.02
	MIMIC-IV	0.66 ± 0.01	0.63 ± 0.01	0.52 ± 0.04	0.74 ± 0.02	0.66 ± 0.01	0.61 ± 0.01	0.58 ± 0.02
	eICU	0.61 ± 0.03	0.56 ± 0.01	0.42 ± 0.11	0.70 ± 0.12	0.60 ± 0.03	0.57 ± 0.01	0.52 ± 0.04
All variables	MIMIC-III	0.87 ± 0.01	0.80 ± 0.01	0.70 ± 0.04	0.90 ± 0.05	0.88 ± 0.05	0.75 ± 0.02	0.78 ± 0.01
	MIMIC-IV	0.88 ± 0.01	0.82 ± 0.01	0.72 ± 0.02	0.92 ± 0.01	0.90 ± 0.01	0.76 ± 0.01	0.80 ± 0.01
	eICU	0.91 ± 0.01	0.83 ± 0.01	0.81 ± 0.03	0.86 ± 0.03	0.85 ± 0.03	0.82 ± 0.02	0.83 ± 0.01

**Table 5:** Performance of the GRU model with different observation windows (T${}_{O}$ = 8/16/24/48).

Variables	Dataset	AUC (Mean ± Standard Deviation)			
		48 h	24 h	16 h	8 h
Vital signs	MIMIC-III	**0.85 ± 0.01**	0.81 ± 0.02	0.80 ± 0.02	0.80 ± 0.00
	MIMIC-IV	**0.87 ± 0.01**	0.85 ± 0.01	0.85 ± 0.01	0.85 ± 0.01
	eICU	**0.89 ± 0.02**	0.87 ± 0.01	0.86 ± 0.02	0.86 ± 0.01
Laboratory variables	MIMIC-III	**0.69 ± 0.02**	0.68 ± 0.01	0.66 ± 0.02	0.66 ± 0.03
	MIMIC-IV	**0.66 ± 0.01**	0.65 ± 0.02	0.64 ± 0.02	0.63 ± 0.02
	eICU	**0.61 ± 0.03**	0.56 ± 0.02	0.56 ± 0.01	0.57 ± 0.01
All variables	MIMIC-III	**0.87 ± 0.01**	0.85 ± 0.01	0.84 ± 0.01	0.84 ± 0.01
	MIMIC-IV	**0.88 ± 0.01**	0.87 ± 0.01	0.87 ± 0.01	0.87 ± 0.00
	eICU	**0.91 ± 0.01**	0.88 ± 0.01	0.88 ± 0.01	0.88 ± 0.01

**Table 6:** Performance of our 48 h GRU model for each intensive care type on the test set for each critical care database.

ICU Type *	MIMIC-III		MIMIC-IV		eICU	
	AUC ${}^{†}$	N	AUC ${}^{†}$	N	AUC ${}^{†}$	N
CCU: Coronary Care Unit	0.86 ± 0.01	426	0.88 ± 0.01	434	0.91 ± 0.01	425
CSRU: Cardiac Surgery Recovery Unit	0.90 ± 0.01	537	–	–	–	–
MICU: Medical ICU	0.88 ± 0.01	911	0.87 ± 0.01	640	0.91 ± 0.01	483
SICU: Surgical ICU	0.88 ± 0.01	496	0.84 ± 0.01	536	0.91 ± 0.01	393
TSICU: Trauma Surgical ICU	0.87 ± 0.01	383	0.92 ± 0.01	445	–	–
Med-Surg ICU: Medical Surgical ICU	–	–	0.86 ± 0.01	489	0.91 ± 0.01	2681
CTICU: Cardiothoracic ICU	–	–	–	–	0.91 ± 0.01	243
CCU-CTICU: Coronary Care Unit/Cardiothoracic ICU	–	–	–	–	0.92 ± 0.01	449
Neuro ICU: Neurological ICU	–	–	0.91 ± 0.01	52	0.91 ± 0.01	600
Cardiac ICU: Cardiological ICU	–	–	–	–	–	–
CVICU: Cardiac Vascular ICU	–	–	0.87 ± 0.01	562	–	–
CSICU: Cardiac Surgery ICU	–	–	–	–	0.92 ± 0.01	169
**Total**	0.87 ± 0.01	2753	0.88 ± 0.01	3158	0.91 ± 0.01	5443

^*^ ICU types as appeared in the original critical care databases, ^†^ AUC: AUC (mean ± standard deviation).

## Data Availability

The data employed in this study are openly available in the PhysioNet repository: MIMIC-III [11], MIMIC-IV [12] and eICU [13].

## Abbreviations

The following abbreviations are used in this manuscript:

APACHE	Acute physiology and chronic health evaluation
AUC	Area under the receiver operating characteristic curve
BUN	Blood urea nitrogen
Diastolic BP	Diastolic blood pressure
MLP	Multilayer perceptron
GRU	Gated recurrent unit
HCO${}_{3}$	Bicarbonate
FRNN	Fully-connected recurrent neural network
ICU	Intensive care unit
LSTM	Long short-term memory
Mean AP	Mean arterial pressure
MEWS	Modified early warning score
MIMIC	Multiparameter intelligence monitoring in intensive care
NEWS	National early warning score
PTT	Partial thromboplastin time
ROC	Receiver operating characteristic curve
RNN	Recurrent neural network
SAPS	Simplified acute physiology score
SD	Standard deviation
SHAP	SHapley Additive exPlanation
Systolic BP	Systolic blood pressure
SpO${}_{2}$	Peripheral oxygen saturation
WBC	White blood cells
